# Mycoketide: A CD1c-Presented Antigen with Important Implications in Mycobacterial Infection

**DOI:** 10.1155/2012/981821

**Published:** 2012-03-26

**Authors:** Isamu Matsunaga, Masahiko Sugita

**Affiliations:** Laboratory of Cell Regulation, Institute for Virus Research, Kyoto University, 53 Kawahara-cho, Shogoin, Sakyo-ku, Kyoto 606-8507, Japan

## Abstract

*Mycobacterium tuberculosis* and related mycobacteria species are unique in that the acid-fast bacilli possess a highly lipid-rich cell wall that not simply confers resistance to treatment with acid alcohol, but also controls their survival and virulence. It has recently been established that a fraction of the cell wall lipid components of mycobacteria can function as antigens targeted by the acquired immunity of the host. Human group 1 CD1 molecules (CD1a, CD1b, and CD1c) bind a pool of lipid antigens expressed by mycobacteria and present them to specific T cells, thereby mediating an effective pathway for host defense against tuberculosis. The contrasting and mutually complementary functions of CD1a and CD1b molecules in terms of the repertoire of antigens they bind have been well appreciated, but it remains to be established how CD1c may play a unique role. Nevertheless, recent advances in our understanding of the CD1c structure as well as the biosynthetic pathway of a CD1c-presented antigen, mannose-1, *β*-phosphomycoketide, expressed by pathogenic mycobacteria now unravel a new aspect of the group 1 CD1 biology that has not been appreciated in previous studies of CD1a and CD1b molecules.

## 1. Introduction

The human immune system utilizes multiple maneuvers in both innate and adaptive phases of host defense to fight against the deadly pathogen, *M. tuberculosis*. Immunologists may easily realize this by recollecting the fact that studies of immune responses against Mtb resulted in milestone discoveries of key molecules and pathways of the immune system. Lectins, such as DC-SIGN and Mincle, and Toll-like receptors (TLRs), such as TLR2 and TLR9, prominently interact with ligands derived from Mtb, which is profoundly associated with the innate immunity-stimulating activity observed for the Freund's adjuvant. Classical CD4^+^ helper and CD8^+^ cytotoxic T cells bearing the *αβ* T-cell receptor are activated during Mtb infection, and their differentiation into those of a memory type provides a cellular basis for the prototypic hypersensitivity response classified as the delayed-type hypersensitivity or the type IV allergy. Furthermore, T cells that recognize Mtb-derived antigenic compounds also include V*δ*2^+^ *γδ*T cells, group 2 CD1 (CD1d)-restricted natural killer T cells and group 1 CD1 (CD1a, CD1b, and CD1c)-restricted T cells. Activation of many of these T-cell subsets is not limited to Mtb infection and occurs prominently in response to infections with other bacteria, fungi, and viruses. In this respect, it is noteworthy that virtually all group 1 CD1-presented microbial antigens identified after two decades of extensive studies are derived from Mtb or other related mycobacteria species [1], suggesting the possibility that the group 1 CD1 system may function effectively in the interface between the host immunity and the pathogenic mycobacteria. Indeed, mycolic acids, a mycobacteria-specific long-chain fatty-acid species, appear to be accommodated perfectly into the antigen-binding structure by utilizing all the A′, C′, and F′ pockets and the T′ tunnel elaborated in the human CD1b molecule to elicit specific cytotoxic T-cell responses [2].

 Mice and rats, that are highly useful animals for analyzing many aspects of immune responses, have deleted genes-encoding group 1 CD1 proteins, making it difficult to assess their role in host defense against tuberculosis. However, *in vitro* studies as well as analysis of the guinea pig model of human tuberculosis have detected key features of the group 1 CD1 biology. The human group 1 CD1 molecules are expressed constitutively in most dendritic cell subsets, and their expression is induced in macrophages after Mtb infection [3, 4]. Therefore, the two major host cell types for Mtb infection, namely, dendritic cells and macrophages, are capable of expressing group 1 CD1 molecules. Phagocytosed mycobacteria-derived lipidic antigens are sampled by CD1 molecules differentially in CD1a^+^ early-recycling endosomes and CD1b^+^ lysosomes. Subsequently, antigen-bound CD1 molecules are transported to the plasma membrane for antigen presentation to specific T cells. The lipid-specific, group 1 CD1-restricted T-cell population contains potent cytotoxic T cells that recognize Mtb-infected cells and lyse them. Importantly, immunization of guinea pigs with the Mtb-derived lipids confers protection against subsequent challenge with pathogenic mycobacteria [5, 6], and thus, all of these results collectively underscore group 1 CD1-dependent pathways of the acquired immunity against tuberculosis.

 Whereas many biological aspects have been unraveled for CD1a and CD1b, less is known about CD1c. The CD1c molecules are expressed on CD1a^−^  CD1b^−^ B cells as well as dendritic cells and macrophages and can broadly sample endocytic compartments including those only inefficiently sampled by CD1a and CD1b molecules. These immediately implicate a unique role for CD1c in lipid antigen presentation and host defense against tuberculosis. Mannose-1, *β*-phosphomycoketide (MPM) is the only CD1c-presented natural antigen so far identified that is produced by slow-growing (pathogenic), but not rapidly-growing (less pathogenic) mycobacteria. Thus, the clarification of how MPM is synthesized by mycobacteria and how it is recognized by the host immune system sheds light on distinct aspects of human tuberculosis that have never been appreciated in previous studies of CD1a^−^, CD1b^−^ and MHC-restricted T-cell responses. In this paper, we describe the discovery of MPM, its biosynthetic pathway, functions of MPM-specific T cells, and potential impact of mycoketides on diseases.

## 2. Discovery of CD1c-Restricted T Cells and Their Antigens

 The first and the best characterized Mtb-specific, CD1c-restricted T-cell line was reported in 1999 [7]. Whereas most CD1b-restricted T-cell lines that had been established before then were CD4^−^  CD8^−^ (double negative), the CD1c-restricted T-cell line was positive for the expression of CD8*α* and CD8*β* molecules, and thus, the T-cell line was termed “CD8-1.” The CD8-1 T-cell line was obtained by stimulating CD4^−^ T cells derived from the circulation of a healthy subject with Mtb sonicates. Retrospectively, the successful establishment of the T-cell line appears almost miraculous considering the low precursor frequency of such T cells in uninfected individuals and extremely low abundance of the antigen recognized by CD8-1 (discussed below). As the surface expression of CD8*α* and CD8*β* heterodimers implies, the CD8-1 T cells are those containing intracellular cytotoxic granules [8]. Upon interaction with Mtb-infected dendritic cells, the T cells released granulysin and lyse both the infected host cells and the intracellular microbes. Furthermore, the T cells produced IFN-*γ*, suggesting their role in host defense against tuberculosis.

**Figure 1:**
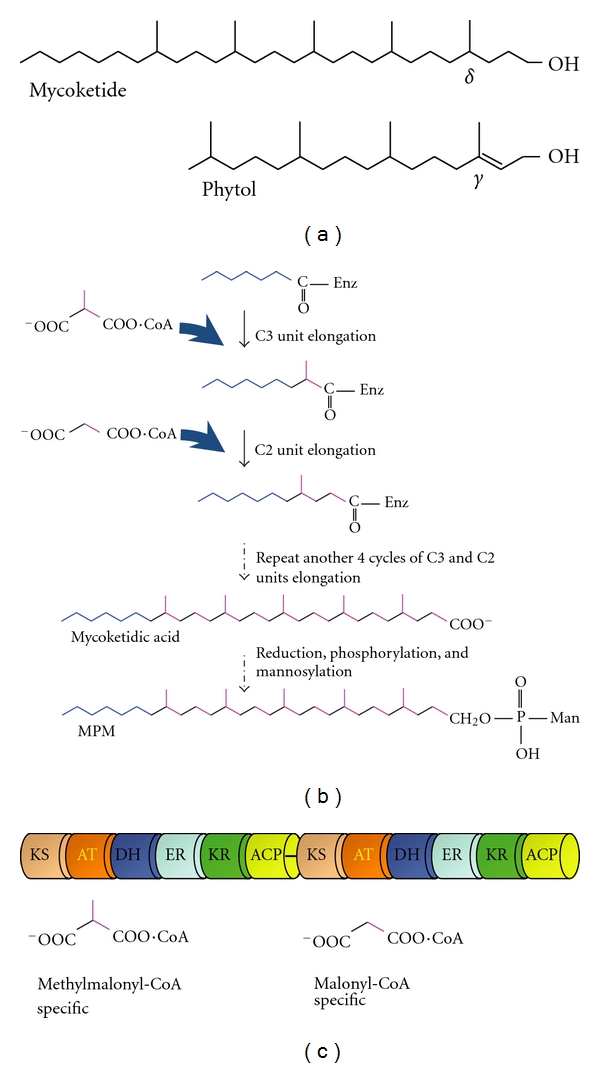
Biosynthesis of MPM. (a) Structures of mycoketide and phytol. Note that the first methyl branch of mycoketide is positioned at the *δ*-carbon whereas that of phytol, an isoprenoid compound, is located at the *γ*-carbon of phytol. (b) A predicted biosynthetic pathway of MPM. The chain elongation of mycoketide occurs on the Pks12 enzyme, followed by its release mediated by a yet unidentified hydrolase enzyme. (c) Schematic structure of Pks12 enzyme. Pks12 contains two tandemly aligned sets of catalytic domains (KS: ketosynthase; AT: acyltransferase; DH: dehydrogenase; ER: enoyl reductase; KR: ketoreductase; ACP: acyl carrier protein). The first set functions for a C3 unit elongation using methylmalonyl-CoA as a substrate and the second set for a C2 unit elongation using malonyl-CoA, which is controlled by the substrate specificities of the AT domains. Note that, unlike FAS enzymes, Pks12 lacks thioesterase domains.

 Identification of the antigen recognized by CD8-1 turned out to be a challenging task because its expression level in mycobacteria appeared very low (less than 1 ppm of dry weight of the bacilli), which contrasted sharply with other CD1-presented antigens [9]. By combining a series of lipid fractionations and a sensitive bioassay with CD8-1, Moody et al. [10] finally determined the structure of the CD1c-presented, *M. avium*-derived antigen. The antigenic compound contained a long-chain alcohol with 5 methyl branches at every 4 carbons, which was coupled with a phosphorylated mannose. This is a reminiscent of mannosyl phosphopolyprenol (MPP), a carrier of sugar moieties utilized in bacteria. Since the polyprenol compound is a polymer of isoprene units, this CD1c-presented antigen was initially designated “mannose phosphoisoprenoid (MPI).” In fact, MPP compounds were able to stimulate CD8-1 although their antigenic activity was less prominent than the natural antigen. Using the MPP compound with a C_35_ alkyl chain as a reference antigen, they detected the circulating CD1c-restricted, antigen-specific T cells in active tuberculosis patients, but not in healthy individuals. Nevertheless, the C_35_ MPP is a compound that is not found in Mtb, and there was an enigma regarding the molecular classification of the CD8-1 antigen as isoprenoid. A known natural compound that is most comparable to the alkyl chain of MPI is phytol, an isoprenoid derivative in plant chlorophyll. Whereas the methyl branch closest to the hydroxyl group of the “isoprenoid” backbone of MPI is placed at the *δ*-position, natural isoprenoids always have this first methyl branch at the *γ*-position (Figure 1(a)). The presence of this extra one carbon can never be accounted for by the biosynthetic pathway of isoprenoids.

## 3. Biosynthesis of MPM

 The “extra-one-carbon” issue pointed to the fact that the alkyl backbone of the CD8-1 antigen was biosynthesized via totally different mechanisms. The later study [9] smartly postulated a sequential and repeated elongation pattern of a C3 carbon unit and a C2 carbon unit, in which the C3 unit adds the methyl branch to the elongation site of the carbon chain, followed by the addition of the C2 unit (Figure 1(b)). Five cycles of this chain elongation sequence would result in the formation of the *δ*-methyl group and the methyl branches sticking out at every 4 carbons of the fully saturated alkyl backbone. This chain elongation mechanism was predicted for the specific polyketide synthase (Pks)-catalyzing reactions, and mutated Mtb strains with the loss of the Pks12 enzyme failed to produce the CD8-1 antigen [9]. These results provided direct evidence that the alkyl backbone of the CD8-1 antigen should be classified not as isoprenoid but as polyketide, and therefore, this polyketide backbone produced by mycobacteria was renamed “mycoketide”.

 The Pks12 enzyme is a huge multifunctional polypeptide (~430 kDa) containing two complete sets of fatty acid synthase (FAS)-like catalytic domains. These include ketosynthase (KS), acyltransferase (AT), dehydrogenase (DH), enoyl reductase (ER), ketoreductase (KR), and acyl carrier protein (ACP) domains (Figure 1(c)), all of which are indispensable for the elongation process of the mycoketide chain. As predicted by the elongation mechanisms postulated above, the AT domain located closer to the N-terminus of the Pks12 enzyme utilizes a methylmalonyl-CoA substrate for the C3 unit elongation, whereas the other AT domain favors a malonyl-CoA substrate for the C2 unit elongation [9, 11].

 After 5 cycles of C3 and C2 chain elongation, the constructed alkyl chain is released from the enzyme by hydrolysis, resulting in generation of a carboxylic acid, termed mycoketidic acid (Figure 1(b)). The mycoketidic acid is metabolized by a reduction reaction to the corresponding long-chain alcohol, mycoketide, which is finally phosphorylated and mannosylated to generate MPM. None of the enzymes that catalyze the hydrolysis, reduction, phosphorylation, and mannosylation steps have been identified, but it is predictable, based on the structural properties shared between mycoketide and isoprenoid, that the mannose transferase mediating the biosynthesis of MPP may also function for the MPM synthesis.

## 4. Differential Expression of MPM and Pks12 among Mycobacteria Species

 The CD8-1-reactive MPM can be detected in several strains of Mtb (H37Rv, H37Ra, and CDC1551), *M. bovis* Bacillus Calmette-Guerin (BCG), and *M. avium*, but not in other mycobacteria (*M. smegmatis*, *M. phlei*, and *M. fallax*) [9]. In addition, strain-specific structural variations have been noted for mycoketides. For example, the H37Rv strain produces MPM with C_32_ mycoketide, predominantly, but the CDC1551 strain mainly synthesizes C_30_ mycoketide although these variations are not critical for presentation by CD1c molecules. Besides the MPM-expressing mycobacteria strains listed above, *pks12* genes have also been found in other Mtb strains (F11 and KZN1435), *M. bovis*, *M. africanum*, *M. canetti*, *M. marinum*, *M. ulcerans*, and *M. avium* subsp. *paratuberculosis* (Table 1). It should be noted that all the mycobacteria expressing Pks12 are slow growers, while the *pks12* gene has not been found in any of the nonpathogenic rapidly-growing mycobacteria so far analyzed, suggesting a potential relevance of Pks12 to virulence.

## 5. Binding of MPM to Human CD1c Molecule

 Since the expression level of MPM produced by mycobacteria is extremely low, MPM purification from cultured bacteria is unpractical for testing its biological functions. The total organic synthesis of MPM was made possible by Crich and Dudkin [12], but the synthetic material was a mixture of MPM molecular species with differential chirality at the methyl-branched 5 stereocenter carbons of the mycoketide. The synthetic MPM exhibited a reduced potential to stimulate the CD8-1 T cells, indicating that T cells could react to MPM only with the authentic chirality. On the basis of the elongation sequence of mycoketide mediated by the Pks12 enzyme, the stereocenter carbons should be derived from methylmalonyl-CoA in the condensation step, predicting that these methyl branches are all-*S* isoform [13]. The chiral synthesis of MPM was achieved by van Summeren et al. [14], and indeed, the all-*S* form of MPM showed a CD8-1 stimulating activity that was comparable with the natural compound.

Success of the total organic synthesis of the authentic MPM has now made the CD1c-presented antigen available in an amount that is sufficient for determining how CD1c captures mycoketide [15]. The overall structure of human CD1c is similar to that of the other CD1 members and the MHC class I molecule, in which *α*1, *α*2, and *α*3 extracellular domains of the heavy chain associate noncovalently with *β*2-microglobulin. The *α*1 and *α*2 domains of the CD1 molecule mainly constitute a ligand-binding platform with two ligand-binding cavities, termed A′ and F′ pockets. The high degree of hydrophobicity achieved on the inner surface of these cavities favors interaction with a hydrophobic alkyl chain of the lipid ligands. The crystal structure of the MPM-bound CD1c molecule indicated that the ligand could be accommodated stably in the A′ pocket with the 2nd, 3rd, 4th and 5th methyl groups significantly contributing to the most suitable positioning of MPM in the A′ pocket.

 Unlike the other CD1 molecules, the F′ cavity constructed in the CD1c protein opens to the solvent, and thus, it is not a pocket but a groove. Although the precise role of the F′ structure in ligand binding is unclear, presence of two hydrophobic cavities indicates that CD1c molecules have a potential for binding a ligand with two alkyl chains. In fact, sulfatide, an endogenous lipid in humans, has been shown to be captured by CD1c and presented to specific T cells isolated from multiple sclerosis patients [16]. Recently, a CD1c-restricted T-cell clone was established from a human immunodeficiency virus (HIV)-infected individual that reacted to an N-acylated 12-mer peptide (lipo-12) [17]. The peptide sequence of the lipo-12 antigen partially matched with that of the HIV Nef protein, but an amino acid modification (presumably, tryptophan to kynurenine) was detected at the center of the peptide. In addition, the acyl chain attached to the N-terminus of the peptide was a stearoyl group (C18), which contrasted sharply with the N-myristoylated genuine Nef protein. Since the cavities of CD1c are proposed to interact only weekly with a straight alkyl chain that lacks methyl branches [15], it is presumed that reinforcing molecular interactions may exist between the lipo-12 and the CD1c protein, leading to a model proposed by Scharf et al. [15], in which the peptide portion of lipo-12 penetrates through the F′ channel of the CD1 protein. In support of this assumption, MPM and lipo-12 showed comparable affinities to the CD1c protein, but the reduction of the peptide length of lipo-12 from 12-mer to 6-mer resulted in diminished affinity.

## 6. Proposed Biological Functions of Mycoketides

 Several lines of evidence obtained from studies with *pks12* mutated microbes have pointed to the possibility that mycoketides might function as virulence factors. An initial report indicated that Mtb virulence was reduced by the *pks12* gene disruption in a mouse infection model [18]. However, this mutant appears to have lost the expression of a known virulence factor, phthiocerol dimycocerosates, simultaneously in a *pks12-*independent manner [9]. In *M. marinum*, disruption of *pks12* gene by the transposon mutagenesis also resulted in the attenuation of the microbe in gold fish [19]. In both studies with Mtb and *M. marinum*, careful interpretation of the data would be required until appropriate complementation experiments are performed. Separately, recent evidence has suggested that the *pks12* gene of the *M. avium* 104 strain is involved in the virulence of the microbe in mice [20]. In this study, the gene complementation resulted in recovery of its virulence, and a role for the Pks12 protein in suppressing phagosomal acidification was proposed [20]. These results as well as the fact that the *pks12* gene is conserved only in slow-growing pathogenic mycobacteria strongly indicate that mycoketides may significantly control their virulence. Nevertheless, a striking difference exists between mycoketides and other defined virulence factors in terms of their quantities produced by mycobacteria, underscoring totally distinct virulence-controlling mechanisms for mycoketides.

 In addition, disruption of the *pks12* gene in a *M. avium* strain resulted in increased susceptibility to antibiotics functioning against distinct molecular targets [21]. This suggests that the Pks12 protein may contribute to the common drug-transporting capacities through the cell wall that functions in multidrug-resistant *M. avium* complex (MAC).

 One possibility that accounts for these observations is that, by analogy to the structurally related polyprenol, mycoketide may function as a carrier of sugar moieties. The second possibility is that mycoketide may function as a surrogate for the prenyl moiety of isoprenoid compounds, which is known to be utilized during the biosynthesis of menaquinone [22] and carotenoid [23], but this model does not reasonably explain why isoprenoids produced by *pks12*-mutated microbes fail to compensate for the defect. Alternatively, mycoketides may be used for a substitute of farnesyl or geranylgeranyl moieties of the prenylated proteins. Although it has not yet been demonstrated that mycobacteria have their own prenyltransferases, some bacterial proteins are reported to be prenylated by borrowing the host-derived transferases [24]. If this is the case with mycobacteria, some mycobacterial proteins could potentially be “mycoketidated” in the host cells. It would be also possible that the released mycoketide may be used for undesired modification of the host phagosomal proteins with mycoketides, resulting in perturbation of the host defense mechanisms.

 On the basis of the extremely low abundance of mycoketides in mycobacteria, one might also consider the possibility that they might function as bioactive compounds for inducing biological signals. The amount of MPM produced in the standard liquid culture is within the range of ~1 nM concentration, one-third of which is detected outside the bacteria. It should be noted that the Pks12-related metabolites function as lipidic secondary metabolites [25], and that a significant fraction of microbial secondary metabolites can serve as signaling factors even in very low concentrations [26]. Therefore, the released MPM may be detected by a specific sensor of the microbes themselves or the neighboring microbes, transmitting signals that regulate the rate of their cell division, survival; or virulence.

## 7. Conclusions and Perspectives

 Only a few CD1c-presented antigens have been identified by now, and the molecular requirements for their interaction with CD1c and presentation to T cells remain to be fully elucidated. Nevertheless, the crystal structure of the synthetic MPM bound CD1c molecules as well as the identification of a new type of CD1c-presented lipopeptide antigen (lipo-12) have disclosed unique properties that distinguish CD1c from other CD1 molecules. A single alkyl chain with multiple methyl branches fits well into the A′ pocket. By utilizing the opened F′ structure, lipopeptides with a long peptide chain coupled with a single alkyl chain may also sit comfortably in the chair. Like sulfatide, glycolipids with two relatively short carbon chains may also be accepted. Structure-based selection of CD1c ligands from a huge array of mycobacteria-derived lipidic molecules would be a tough task, but molecular modeling with the crystallized CD1c molecules may allow us to identify a pool of CD1c ligands in nonwet experiments. Furthermore, testing T-cell reactivity to these ligands in mycobacteria-infected patients would help us to understand how CD1c-reactive T cells may function to control infections. In this respect, it is noteworthy that the CD1c-presented MPM antigen is expressed primarily in pathogenic mycobacteria, suggesting that MPM-specific, CD1c-restricted T cell could potentially discriminate pathogenic mycobacteria from nonpathogenic environmental mycobacteria.

 The precise function of mycoketides remains to be determined, but it is intriguing to reasonably speculate that they may control the notorious multidrug resistance acquired naturally by MAC. Our pilot study detects a correlation between MPM production and drug resistance in clinically isolated MAC strains, also supporting the hypothesis. Thus, this line of study may raise the possibility that mycoketides may be the new molecular target for antibiotics that can control not only Mtb but also multi-drug resistant MAC.

## Figures and Tables

**Table 1:** Distribution of *pks12* gene in mycobacteria species^a^.

Mycobacteria strains	Gene	Amino acid residues	Identity^b^ (%)	MPM^c^
* M. tuberculosis* H37Rv	Rv 2048c	4151	100	+
H37Ra	MRA_2063	4151	99.9	+
CDC1551	MT2108	4151	99.7	+
F11	TBFG_12085	4151	99.7	n.d.
KZN1435	TBMG_01933	4152	99.4	n.d.
* M. bovis* AF2122/97	Mbo2074c	4151	99.4	n.d.
BCG Tokyo 172	JTY_2062	4151	99.6	n.d.
BCG Pasteur 1173P2	BCG_2067c	4151	99.6	+
* M. africanum* GM041182	MAF_20630	4151	99.9	n.d.
* M. canetti* CIPT140010059	MCAN_20711	4154	99.0	n.d.
* M. marinum *ATCC BAA-535	MMAR_3025	4187	83.1	n.d.
* M. ulcerans* Agy99	MUL_2266	4191	82.7	n.d.
* M. avium* 104	MAV_2450	4171	80.8	+^d^
* M. avium* subsp. *paratuberculosis *k10	MAP1796c	4170	80.4	n.d.

^a^The criteria for identifying the *pks12* gene is that the encoded Pks protein contains two tandem sets of FAS catalytic domains (KS, AT, DH, ER, KR, and ACP) on one polypeptide and that the two AT domains show substrate specificities for methylmalonyl-CoA and malonyl-CoA, respectively (Figure 1(c)). Without any of these, complete mycoketide structure would not be generated. The PKS database (http://www.nii.res.in/nrps-pks.html) is very useful to predict the catalytic domains and substrate specificity of a Pks enzyme.

^b^Identities to the Rv2048c protein in the aminoacid sequences.

^c^The MPM was determined by a bioassay using the CD8-1 T cells (n.d. not determined).

^d^The MPM production was determined with *M. avium* serovar 4 strain (ATCC35767).
